# Head & neck optical diagnostics: vision of the future of surgery

**DOI:** 10.1186/1758-3284-1-25

**Published:** 2009-07-13

**Authors:** Tahwinder Upile, Waseem Jerjes, Henricus JCM Sterenborg, Adel K El-Naggar, Ann Sandison, Max JH Witjes, Merrill A Biel, Irving Bigio, Brian JF Wong, Ann Gillenwater, Alexander J MacRobert, Dominic J Robinson, Christian S Betz, Herbert Stepp, Lina Bolotine, Gordon McKenzie, Charles Alexander Mosse, Hugh Barr, Zhongping Chen, Kristian Berg, Anil K D'Cruz, Nicholas Stone, Catherine Kendall, Sheila Fisher, Andreas Leunig, Malini Olivo, Rebecca Richards-Kortum, Khee Chee Soo, Vanderlei Bagnato, Lin-Ping Choo-Smith, Katarina Svanberg, I Bing Tan, Brian C Wilson, Herbert Wolfsen, Arjun G Yodh, Colin Hopper

**Affiliations:** 1 The "Head and Neck Optical Diagnostics Society" Council, Head & Neck Centre, University College Hospital, 250 Euston Road, London, NW1 2PG, UK; 2 UCLH Head and Neck Centre, London, UK; 3 Department of Surgery, University College London Medical School, London, UK; 4 Unit of Oral & Maxillofacial Surgery, Division of Maxillofacial, Diagnostic, Medical and Surgical Sciences, UCL Eastman Dental Institute, London, UK; 5 Center for Optical Diagnostics and Therapy, Erasmus University Medical Center, Rotterdam, the Netherlands; 6 Department of Pathology, The University of Texas M.D. Anderson Cancer Center, Houston, Texas, USA; 7 Department of Histopathology, Imperial College and The Hammersmith Hospitals, London, UK; 8 Department of Oral & Maxilofacial Surgery, University Medical Center Groningen, the Netherlands; 9 Virginia Piper Cancer Institute-Abbott Northwestern Hospital, Minnesota, USA; 10 Department of Biomedical Engineering, Electrical & Computer Engineering, Physics, Boston University, Boston, USA; 11 The Beckman Laser Institute and Medical Clinic, The University of California Irvine, Irvine, CA, USA; 12 Department of Head and Neck Surgery, Division of Surgery, The University of Texas M. D. Anderson Cancer Center, Houston, TX, USA; 13 National Medical Laser Centre, University College London, London, UK; 14 Center for Optical Diagnostics and Therapy, Department of Radiation Oncology, Erasmus University Medical Center, Rotterdam, the Netherlands; 15 Department of Otorhinolaryngology, Head & Neck Surgery, Ludwig Maximilian University, Munich, Germany; 16 LIFE Center, University Clinic Munich, Munich, Germany; 17 Research Centre for Automatic Control (CRAN), Nancy-University, UMR CNRS, France; 18 Michelson Diagnostics, 11A Grays Farm Production Village, Grays Farm Road, Orpington, Kent, BR5 3BD, UK; 19 Gloucestershire Hospitals NHS Foundation Trust, Gloucester, UK; 20 Department of Biomedical Engineering, Beckman Laser Institute, University of California, Irvine, USA; 21 Dept. of Radiation Biology, The Norwegian Radium Hospital, Montebello, Norway; 22 Department of Oral & Maxillofacial Surgery, Tata Memorial Hospital, Mumbai, India; 23 Department of Oral & Maxillofacial Surgery, Leeds Dental Institute, Leeds, UK; 24 Photodynamic Therapy and Diagnosis Laboratory, Division of Medical Sciences, National Cancer Centre, Singapore; 25 Department of Bioengineering, Rice University, Houston, USA; 26 National Cancer Centre, Singapore 169610, Singapore; 27 Univiersity of Sao Paulo, Sao Carlos, SP, Brazil; 28 National Research Council Canada-Institute for Biodiagnostics, Winnipeg, Manitoba, Canada; 29 Division of Oncology, Lund University Hospital, Lund, Sweden; 30 Department of Head & Neck Oncology & Surgery, The Netherlands Cancer Institute – Antoni van Leeuwenhoek Hospital, Amsterdam, the Netherlands; 31 Division of BioPhysics and BioImaging, Ontario Cancer Institute, Ontario, Canada; 32 Department of Medical Biophysics, Faculty of Medicine, University of Toronto, Toronto, Canada; 33 Division of Gastroenterology and Hepatology, Mayo Clinic, Florida, USA; 34 Physics and Astronomy, University of Pennsylvania, Philadelphia, USA

## Abstract

Review paper and Proceedings of the Inaugural Meeting of the Head and Neck Optical Diagnostics Society (HNODS) on March 14^th ^2009 at University College London.

The aim of our research must be to provide breakthrough translational research which can be applied clinically in the immediate rather than the near future. We are fortunate that this is indeed a possibility and may fundamentally change current clinical and surgical practice to improve our patients' lives.

## Introduction

Upper aero-digestive tract (UADT) carcinomas continue to be the 6^th ^most common cancer worldwide with approximately 270,000 new oral cavity tumours per year [1]. Unfortunately, the majority of these tumours present in late stage with the attendant functional, psychological and economic costs to their victims. Early diagnosis is often delayed as tumour precursors or early cancers are hardly visible and not picked up by common imaging methods. It's clearly evident that screening and early detection of the cancer and its early precursors have the potential to reduce the morbidity and mortality of this disease. In that context, current oral examination methods including incandescent light or toluidine blue, reflectance visualization and illumination with chemi-luminescent light source, are largely subjective, dependent on the experience of the examiner and are considered in-effective tools in primary care settings [2].

The true or important surgical margin of these lesions is still not defined [3]. In the treatment of cancer the fundamental surgical goal is to remove all local malignant disease and leave no residual malignant cells. Studies have demonstrated the benefit of achieving negative resection margins in terms of disease free local recurrence and overall survival. The surgical margins for head & neck cancer may vary widely depending on the site of disease. This variation reflects the biological and anatomical environment of the tumour site at macroscopic and microscopic levels. There is no accepted standard for the quantity of normal tissue to be removed and the effect of positive margins on recurrence rate appears to be considerably dependent on the site of the tumour. The extent of tumour volume resection is determined by the need for cancer control and the peri-operative, functional and aesthetic morbidity of the surgery.

Resection margins are currently assessed intra-operatively by frozen section and retrospectively after definitive histological analysis of the resection specimen. There are limitations to this assessment. The margin may not be consistent in three dimensions and may be susceptible to errors in sampling and histological interpretation. Assigning the true excision margin may be difficult due to post-excision changes secondary to shrinkage and fixation [3].

Local recurrence occurs even among tumours with extensive histological demonstration of adequate resection margins. Sites with significant recurrence rates after negative resection margins are oral cavity, sub-mandibular region, tonsil and pharynx. Therefore, it is accepted that cancers at these sites require larger margins of excision than tumours elsewhere in the head and neck [3].

**Figure 1:**
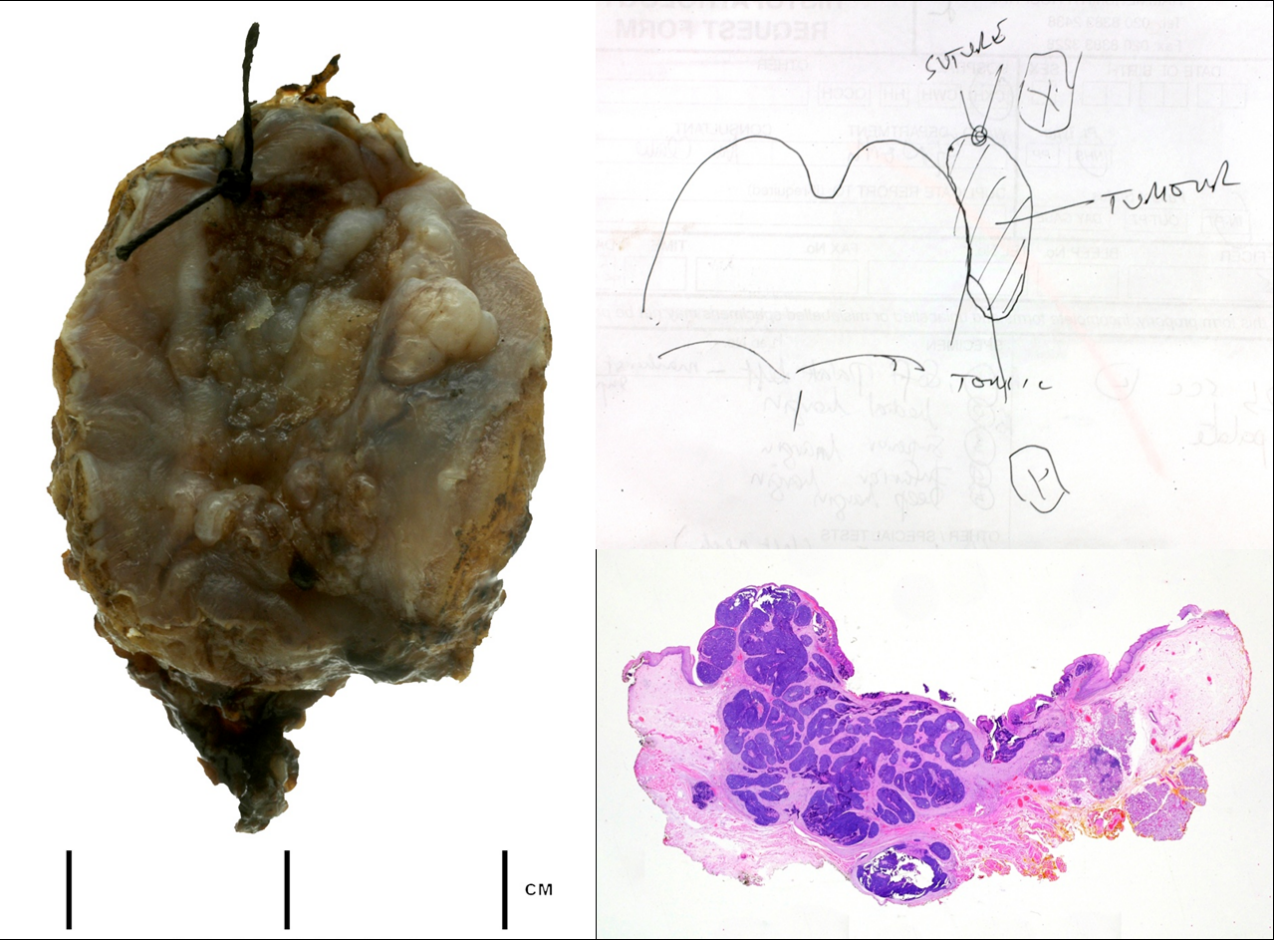
**Macroscopic view of laser resected tumour orientated by suture and clinical diagram**. This shows the complexity of pathological interpretation which can be liable to sampling error. A whole mount view of H&E stained section of transverse slice through the tumour and tonsil show the close margin of excision. It is reasonable to assume that high quality 'real-time' pathological data would aid surgical incision and ensure a more complete excision. Retrospective analysis paraffin section H&E appears less useful since it cannot immediately inform surgery only later therapy. Optical diagnostics technology may provide a means to improve surgical treatment and eventual outcome by informing the surgeon in 'real-time' and improving the margin; (Courtesy of Dr A Sandison, Imperial College, London).

The development of optical techniques for non-invasive diagnosis of disease is an ongoing challenge to biomedical optics. Optical diagnostics have proved to be a reliable resource that can be used to give an instant diagnosis of soft and, more recently, hard tissue diseases. In the field of head and neck malignancy, most of the experimental spectroscopy work has been performed using fluorescence spectroscopy, Raman spectroscopy, elastic scattering spectroscopy, microendoscopy and optical coherence tomography [4]. Furthermore, the exponential rise in computer processing power, adaptive statistical software packages and the development of nanofilament and refined fibre optics has lead to quantum leaps in our ability to define the edge of pathology. The search for the optimum imaging modality to refine this "disease edge" is occurring along several often mutually complementary optical technological pathways which were outlined at the meeting and are critically summarised below (Figure 1).

Tumour margin detection in real-time without the use of additional molecular stain would be desirable. Surgical use of a microscopy tool would be ideal, but most systems focus on microscopic disease evaluation, whereas tools for macroscopic scanning of tissue such as enhanced endoscopy imaging are less well developed.

For head and neck cancer there are two immediate fields of potential application

1) Screening for second primaries in patients with a history of cancer. This requires imaging techniques or an approach where a larger area can be scanned quickly.

2) Distinguishing potentially malignant visible primary lesions from benign ones. Here fibre-optic point measurements can be used as the location of the lesion is known.

## Techniques

### Elastic Scattering Spectroscopy

Optical spectroscopy mediated by fibre-optic probes can be used to perform non-invasive, or minimally-invasive, real-time assessment of tissue pathology *in situ*. The method of elastic scattering spectroscopy (ESS) is sensitive to the sub-cellular architectural changes, such as nuclear grade and nuclear to cytoplasm ratio, mitochondrial size and density...etc., which correlates with features often used by pathologists when performing histological assessment. ESS is also sensitive to ultrastructural changes that are beyond the Abbe resolution limit for optical microscopy, and thus conveys information that may not be available from conventional histology. ESS has proved to be a promising method for detecting premalignant and malignant changes in a variety of organ areas, including oral tissues, with high sensitivity and specificity. Several head and neck tissues, including lymph nodes, archival bones, skin and resection margins, have been interrogated using ESS with very promising results [4,5].

**Figure 2:**
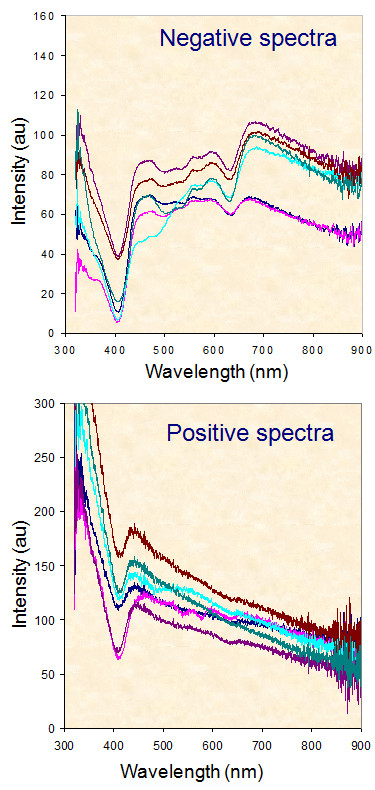
**ESS spectra obtained from bivalved cervical lymph nodes showing spectra acquired from histopathologicaly negative nodes (top) and positive ones (bottom); (Courtesy of Dr W Jerjes, University College London, London)**.

It should be noted that the ESS method senses micromorphology changes without actually imaging the microscopic structure. Consequently, diagnosis is provided by objective statistical and analytical methods, rather than subjective interpretation of images. Clinical studies of ESS have been conducted in a variety of organ sites, and larger-scale clinical studies are now ongoing. Further developments include an analytical model that extracts, from the ESS spectra, the underlying physical correlates of the tissue relating to disease, such as mean size of scattering centres, blood perfusion and haemoglobin oxygen saturation (Figure 2).

### Differential path-length spectroscopy (DPS)

**Figure 3:**
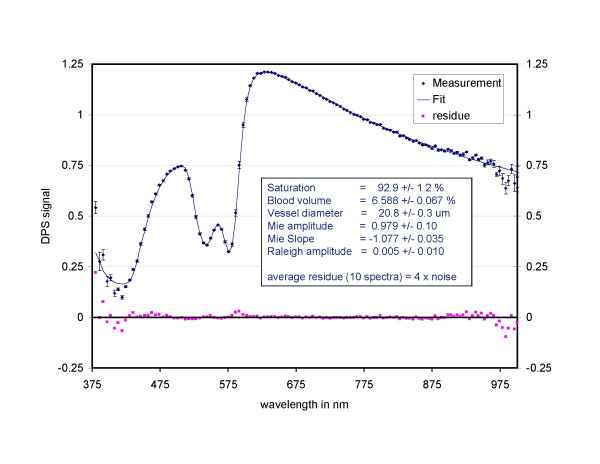
**DPS spectrum of normal oral mucosa with a fit of the descriptive model**. The graph indicates that the fit residues are in the order of the measurement noise. With the parameters derived we can classify a measurement site where the overall amplitude of scattering, the Mie amplitude, the saturation, the vessel diameter and the blood vessel to blood volume ratio contribute significantly to the classification; (Courtesy of Prof HJCM Sterenborg, Erasmus Medical Center, Rotterdam).

Various techniques for point measurements have been developed and investigated clinically for different applications. Differential path-length spectroscopy is a recently developed fibre-optic point measurement technique that measures scattered light in a broad spectrum. Due to the specific fibre-optic geometry, only scattered photons that have travelled a predetermined path length are measured. The spectrum is analyzed mathematically and the measured curve is translated into a set of parameters that are related to the microvasculature and to the intracellular morphology. DPS has been extensively evaluated on optical phantoms and tested clinically in various clinical applications (Figure 3) [6].

The first measurements in biopsy proven squamous cell carcinoma showed significant changes in both vascular and morphological parameters. Measurements on thick keratinized lesions however failed to generate any vascular signatures. This is related to the sampling depth of the standard optical fibres used. Recently the group developed a fibre-optic probe with a ~1 mm sampling depth. Measurements on several leukoplakias have shown that with this new probe one may now sample just below the keratin layer to obtain vascular signatures. This enables clinically significant diagnostic measurements [6].

### Spectral scatter scanning system

A novel technique showing great translational utility is a recent raster scanning scatter spectroscopy system that has been evaluated for imaging the spectral signature remitted from tissue, with real-time classification algorithm, which maximizes the ability to identify regions of tumour from regions of normal tissue. The system uses a wide band of wavelengths from 400 nm up to 700 nm, and recovers the scatter power, scatter amplitude, and absorption species, from the reflectance from a 100 micron spot, allowing imaging of tissue a high frame rate. The system uses dark field illumination and spectrometer detection in the emission channel together with a scanning mirror. The early prototypes of the system were tested on pancreas tumours and prostate tumour margin detection, and current work is ongoing in breast cancer margin delineation [7].

### Raman Spectroscopy

**Figure 4:**
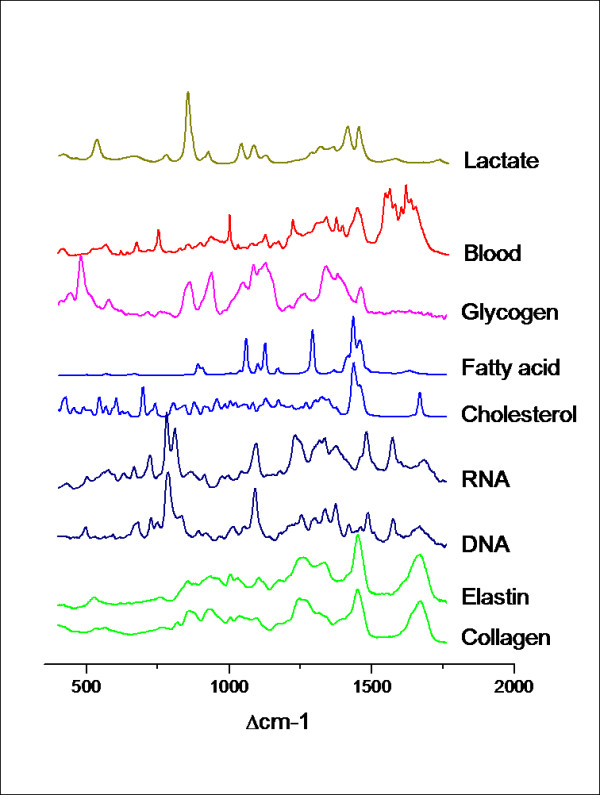
**Examples of molecules and their Raman spectrum; (Courtesy of Dr GW Puppels, Erasmus Medical Center, Rotterdam)**.

Raman spectroscopy is a spectroscopic technique used in physics and chemistry to study vibrational and other low-frequency modes in a system. Raman spectroscopy is laser-based technique that enables chemical characterization and structure of molecules in sample. Raman spectroscopy methods are being considered as techniques which could be complementary or even alternative to biopsy, pathology and clinical assays in many medical technologies (Figure 4).

The applications of Raman spectroscopy in the life sciences are still in the early stages of development. Raman spectroscopy is being investigated in a broad spectrum of biological and toxicological sciences. In oncology Raman is being investigated as a diagnostic tool for characterising cancer cells and early malignant changes and distinguishing these from normal cells. Raman spectroscopy has the distinct advantage over other optical techniques that it provides information on molecular composition and structure of living tissue. There is a strong rationale for using Raman spectroscopy in epithelial cancer. Although Raman spectroscopy has been investigated for several decades, clinical head & neck studies are scarce [8].

A significant problem associated with Raman applications arises from inherently weak signal produced by the Raman Effect. Biomedical samples are extremely intricate systems which reflect complex Raman spectra. Raman bands due to biological constitutes are generally overlapped, making it difficult to identify individual components correctly. Furthermore, due to the minimal sample preparation encountered in the clinical environment, biomedical sample samples usually produce a strong fluorescent background which may completely obscure the true Raman signals [8].

### Fluorescence Techniques

In an attempt to improve intra-operative tissue diagnosis, tools and methods for an "optical biopsy" have been proposed, some of them exploiting fluorescent properties of endogenous or exogenous fluorochromes [9,10].

Fluorescence spectroscopy tries to capture characteristic spectral features of fluorochromes and correlate these with the disease state. Several mathematical methods have been proposed to evaluate recorded spectra to maximize the discrimination between "normal" and "malignant".

Fluorescence imaging aims at highlighting malignant tissue, especially where it is not evident under white light in a large field of view. Autofluorescence as well as drug-induced fluorescence can be detected and displayed with commercial equipment. They usually rely on capturing fluorescence in one or two colour channels and remission in another channel. Sophisticated image processing to quantify fluorescence or eliminate disturbing signal is only slowly becoming available.

**Figure 5:**
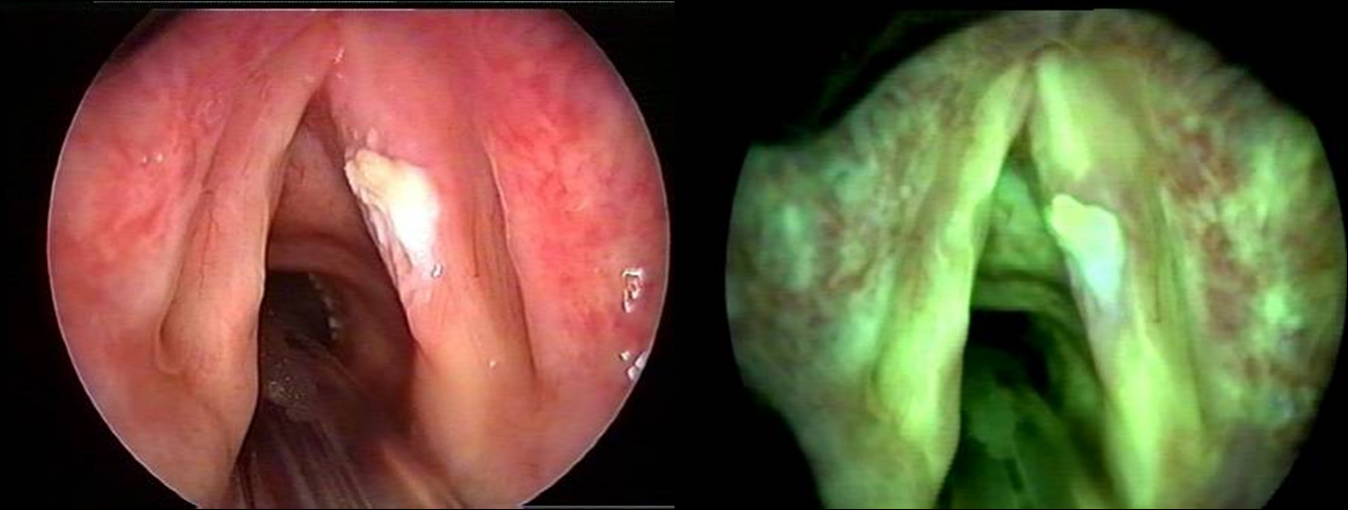
**Representative enhanced fluorescence image of a T1 SCC of the vocal cords; (Courtesy of Dr CS Betz, Ludwig Maximilian University, Munich)**.

Auto-fluorescence imaging has recently been shown to improve the detection of premalignant and malignant oral lesions. This method is based on the illumination in the absorption of tissue fluorophore molecules (NADH and FAD in the epithelial layer and collagen, and elastin in the stroma) in ultraviolet visible spectrum leading to the emission of lower energy photon that can be detected as fluorescence from the oral surface mucosa. Studies of these methods in normal oral mucosa have shown increased green fluorescence in comparison to neoplastic lesions upon ultraviolet (UV) or near UV light source. The histopathological manifestations and heterogeneity of oral squamous lesions and the confounding factors for the validation and the clinical applications of auto-fluorescence imaging are hurdles to overcome [9,10] (Figure 5).

The fluorescence contrast is even slightly enhanced by using exogenously applied fluorescent markers or their precursors (e.g., 5-aminolevulinic acid induced Protoporphyrin IX). Recent advances include the possibility to extract true spectra of single fluorophores ("intrinsic spectra") by mathematically eliminating the undesired influences of scattering and absorption. As well, tumour-specific enzymes are about to be specifically targeted by fluorescent markers "smart probes" in order to improve both sensitivity and specificity.

### Optical Coherence Tomography

**Figure 6:**
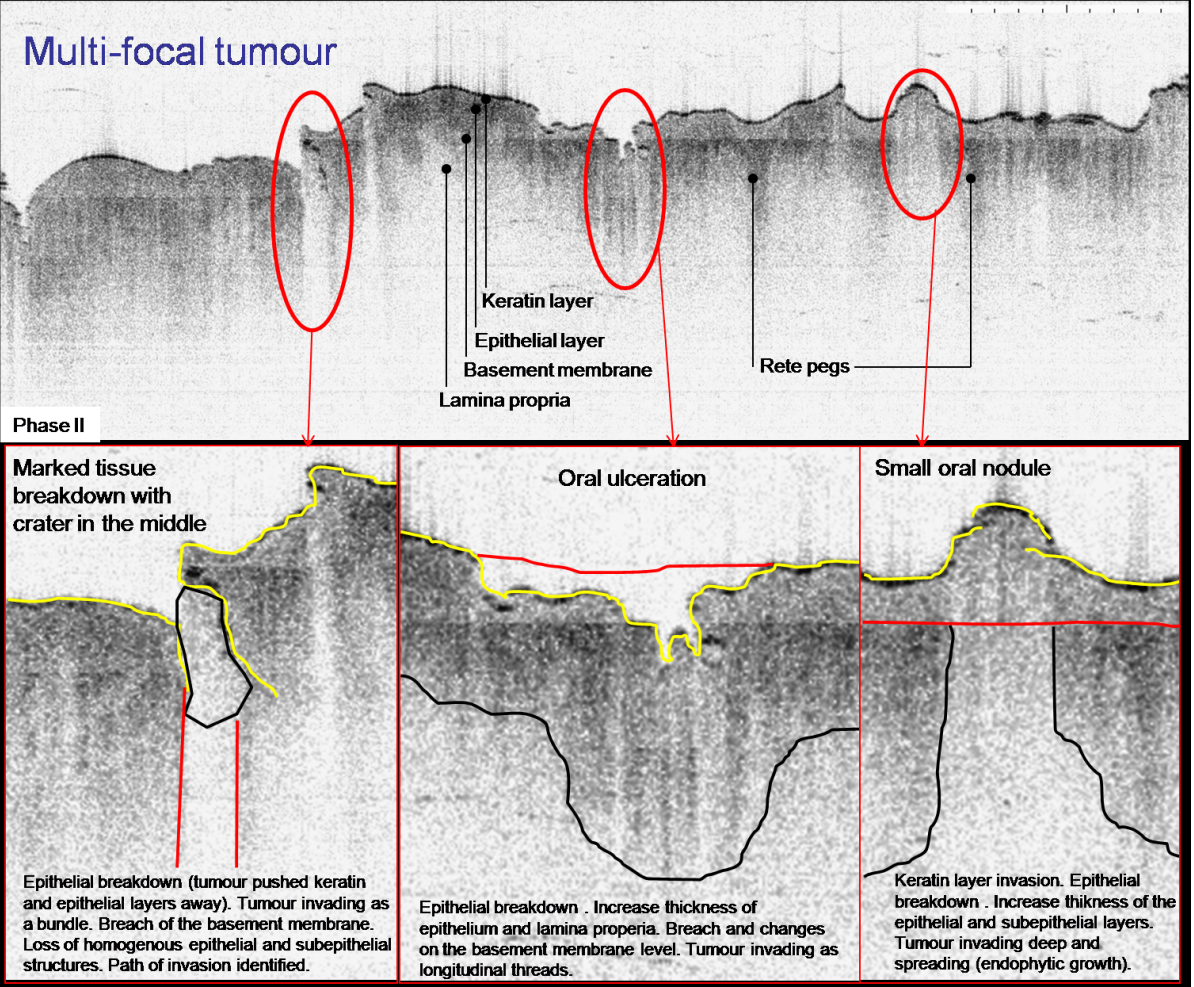
**An inverted OCT image of the lateral border of the tongue**. There are processing artefacts running across the image. The surface differentiation is evident as visible tongue papillae poorly. 'Rete Ridges/pegs' can be seen projecting into the underlying mucosa. The various forms of tongue papillae are also visible. Histologically this area was found to represent multifocal squamous cell carcinoma; (Courtesy of Drs W Jerjes and Z Hamdoon, University College London, London)

Optical coherence tomography is an imaging modality that uses light to determine cross-sectional anatomy in turbid media such as living tissues. Optical coherence tomography (OCT) is based on coherence-domain optical technology. OCT takes advantage of the short coherence length of broadband light sources to perform micrometer-scale, cross-sectional imaging of biological tissue. OCT is analogous to ultrasound imaging except that it uses light rather than sound. The high spatial resolution of OCT enables non-invasive *in vivo *"optical biopsy" and provides immediate and localized diagnostic information (Figure 6).

Despite the recent development of Fourier domain OCT that significantly increases imaging speed and sensitivity, the OCT system that achieves both high speed and high sensitivity simultaneously at 1.3 μm is not currently available. The recent development of a Fourier-domain-mode-lock (FDML) swept source based OCT system can now achieve high speed (>100 kHz A-scan rate) and high spatial resolution (<4 μm) simultaneously. In addition, the development of various miniature scanning probes that allow high-speed 3-D OCT imaging will be reported. This has been augmented by the development of a non-iterative digital focusing method to alleviate the compromise between lateral resolution and depth measurement range, which allows high lateral resolution over the full depth measurement range [11].

The major clinical applications of OCT in head and neck surgery that have been explored recently are: examination of the true vocal folds with the aim of identifying and characterizing pre-cancerous and early stage malignancy and examination of the paediatric/neonatal sub-glottis. OCT imaging can discern subtle differences in the sub-glottic mucosa and hopefully provide a means to identify patients at risk for extubation failure, and ideally in the future be used in the neonatal ICU to optimize endotracheal tube management [12].

This technology still as yet gives the unfulfilled promise of the basement membrane recognition which is all important in defining the extent of malignant spread and disease prognosis. Its resolution is still clearly just below the tissue level but rapidly approaching the cellular scale. Further developments in hardware (i.e. optics, probe design and laser scanning) and software processing (including pattern recognition and rendering) continue to improve resolution and clinical utility.

### Other techniques

Several other optically based systems for tissue interrogation are described as well as their current ex-vivo and practical applications

### Infrared Spectroscopy

The potential role of infrared spectroscopy in biomedical science has been described to distinguish different biomolecules by probing chemical bond vibrations and using these molecular and sub-molecular patterns to define and differentiate pathological from healthy samples. Several protocols have been described to exploit the potential of infrared spectroscopy in defining spectral profiles in salivary gland disease attributable to various kinds of cancer and the corresponding healthy tissues. Researchers suggest the potential of infrared micro-spectroscopy imaging, in combination with multivariate data analysis, to highlight even subtle biochemical and morphological changes, distinguishing various kinds and grades of neoplasia in human tissues [13].

### Con-focal endo-microscopy

Confocal Endomicroscopy (CEM) is a non invasive imaging tool enabling "optical biopsies" of tissues at cellular level. Clinical studies have successfully reported the accuracy of CEM for the characterization of gastrointestinal, dermatologic and ocular diseases. Researchers have assessesed the potential use of endomicroscopy in combination with clinically approved fluorophores to characterize premalignant and malignant lesions in human larynx. Imaging of squamous cell carcinoma provided clear information on the heterogeneous distribution of tumour cells surrounded by stroma. Cellular anomalies and disorders of keratinisation such as dyskeratosis and keratin pearls were also discerned by CEM and the images corroborated with histological data [14].

## Conclusion

Optical diagnosis of the head and neck is a rapidly developing area of clinical research that can be readily translated to inform patient treatment and overall quality of life. Much still needs to be achieved and granting organisations are directed to pay attention to this specialty where relatively small investments may lead to enormous dividends in terms of improvements in treatments throughout the fields of medicine and surgery.

## Competing interests

The authors declare that they have no competing interests.

## Authors' contributions

TU, WJ, HJCMS, AKE, AS, MJHW, MAB, IB, BJFW, AG, AJM, DJR,CSB, HS, LB, GM, CAM, HB, ZC, KB, AKD, NS, CK, SF, AL, MO, RR, KCS, VB, LC, KS, IBT, BCW, HW, AGY,CH: contributed to conception and design, designed the review, carried out the literature research, and manuscript preparation, editing and manuscript review. All authors read and approved the final manuscript.
